# Association of Dual *LRRK2 *G2019S and *GBA* Variations With Parkinson Disease Progression

**DOI:** 10.1001/jamanetworkopen.2021.5845

**Published:** 2021-04-21

**Authors:** Roberto A. Ortega, Cuiling Wang, Deborah Raymond, Nicole Bryant, Clemens R. Scherzer, Avner Thaler, Roy N. Alcalay, Andrew B. West, Anat Mirelman, Yuliya Kuras, Karen S. Marder, Nir Giladi, Laurie J. Ozelius, Susan B. Bressman, Rachel Saunders-Pullman

**Affiliations:** 1 Department of Neurology, Mount Sinai Beth Israel, and Icahn School of Medicine, Mount Sinai, New York, New York; 2 Department of Epidemiology and Population Health, Albert Einstein College of Medicine, Yeshiva University, Bronx, New York; 3 Department of Neurology, Albert Einstein College of Medicine, Yeshiva University, Bronx, New York; 4 Duke Center for Neurodegeneration and Neurotherapeutics, Duke University, Durham, North Carolina; 5 Center for Advanced Parkinson Research and Precision Neurology Program, Harvard Medical School, Brigham and Women's Hospital, Boston, Massachusetts; 6 Laboratory for Early Markers of Neurodegeneration, Center for the Study of Movement, Cognition, and Mobility, Neurological Institute, Tel Aviv Medical Center, Sackler School of Medicine, Sagol School of Medicine, Tel-Aviv University, Tel-Aviv, Israel; 7 Department of Neurology, Columbia University Vagelos College of Physicians and Surgeons, New York, New York; 8 Department of Neurology, Massachusetts General Hospital, Boston

## Abstract

**Importance:**

Despite a hypothesis that harboring a leucine-rich repeat kinase 2(*LRRK2*) G2019S variation and a glucocerebrosidase (*GBA*) variant would have a combined deleterious association with disease pathogenesis, milder clinical phenotypes have been reported in dual *LRRK2* and *GBA* variations Parkinson disease (PD) than in *GBA* variation PD alone.

**Objective:**

To evaluate the association of *LRRK2* G2019S and *GBA* variants with longitudinal cognitive and motor decline in PD.

**Design, Setting, and Participants:**

This longitudinal cohort study of continuous measures in *LRRK2* PD, *GBA* PD, *LRRK2*/*GBA* PD, and wild-type idiopathic PD used pooled annual visit data ranging from 2004 to 2019 from the Mount Sinai Beth Israel, Parkinson Disease Biomarker Program, Harvard Biomarkers Study, Ashkenazi Jewish-*LRRK2*-Consortium, Parkinson Progression Marker Initiative, and SPOT-PD studies. Patients who were screened for *GBA* and *LRRK2* variations and completed either a motor or cognitive assessment were included. Data were analyzed from May to July 2020.

**Main Outcomes and Measures:**

The associations of *LRRK2* G2019S and *GBA* genotypes on the rate of decline in Montreal Cognitive Assessment (MoCA) and Movement Disorders Society-Unified Parkinson Disease Rating Scale–Part III scores were examined using linear mixed effects models with PD duration as the time scale.

**Results:**

Among 1193 individuals with PD (mean [SD] age, 66.6 [9.9] years; 490 [41.2%] women), 128 (10.7%) had *GBA* PD, 155 (13.0%) had *LRRK2* PD, 21 (1.8%) had *LRRK2*/*GBA* PD, and 889 (74.5%) had idiopathic PD. Patients with *GBA* PD had faster decline in MoCA than those with *LRRK2*/*GBA* PD (B [SE], −0.31 [0.09] points/y; *P* < .001), *LRRK2* PD (B [SE], −0.33 [0.09] points/y; *P* < .001), or idiopathic PD (B [SE], −0.23 [0.08] points/y; *P* = .005). There was a *LRRK2* G2019S × *GBA* interaction in MoCA decline (B [SE], 0.22 [0.11] points/y; *P* = .04), but not after excluding severe *GBA* variations (B [SE], 0.12 [0.11] points/y; *P* = .28). Patients with *GBA* PD had significantly worse motor progression compared with those with idiopathic PD (B [SE], 0.49 [0.22] points/y; *P* = .03) or *LRRK2* PD (B [SE], 0.77 [0.26] points/y; *P* = .004).

**Conclusions and Relevance:**

These findings suggest that longitudinal cognitive decline in patients with *GBA* PD was more severe than in those with *LRRK2*/*GBA* PD, which more closely resembled *LRRK2* PD. This further supports the notion of a dominant association of *LRRK2* on *GBA* in individuals who carry both and raises the possibility of an *LRRK2* × *GBA* interaction. However, the biological basis of a dominant association or interaction is not clear and is apparently contrary to basic investigations. Study of a larger cohort of individuals with severe *GBA* variation is warranted.

## Key Points

**Question:**

What are the associations of concurrent *LRRK2* G2019S and *GBA* variations with clinical progression of Parkinson disease (PD)?

**Findings:**

In this cohort study combining data for 1193 participants with PD from multiple studies, individuals with dual *LRRK2* G2019S and *GBA* variation PD had a slower rate of cognitive decline than those with *GBA* PD alone, and this was not different from individuals with *LRRK2* G2019S PD alone, supporting the notion that there is a dominant association of the *LRRK2* gene in individuals with both variations. There was also a novel statistical interaction between *LRRK2* G2019S and *GBA* variations in cognitive decline.

**Meaning:**

These findings suggest that there was not a convergent deleterious association of *LRRK2* and *GBA* variations in PD progression, as would be expected based on prior cellular studies.

## Teaser

This cohort study examines the associations of *LRRK2* G2019S and *GBA* variants with longitudinal cognitive and motor decline in Parkinson disease.

## Introduction

Variants in the leucine-rich repeat kinase 2 (*LRRK2*; OMIM 609007) and glucocerebrosidase (*GBA*; OMIM 606463) genes are frequent and worldwide genetic contributors to Parkinson disease (PD) susceptibility.^1,2^ *LRRK2* variations occur in approximately 2% of PD overall, and up to 40% of PD in certain ethnic groups, particularly individuals of Ashkenazi Jewish or Arabian Berber descent.^1^ *GBA* variation and risk variants in *GBA* may be present in as many of 10% of PD worldwide and in approximately 15% or greater in PD among individuals of Ashkenazi Jewish descent.^2,3,4^ Wide ranges in conferred disease risk exist based on allelic heterogeneity. The penetrance of *LRRK2* variations (specifically G2019S), or the chance that a carrier of this variation will manifest PD by late adulthood, has been estimated at 26% to 43% and is lower in individuals with *GBA* variations, ranging from 9% to 19%.^5,6,7,8^ Therefore, additional risk factors are clearly at play.^9,10,11^ One potential critical interaction is the association of *GBA* and *LRRK2* variations in carriers of both variations (hereafter, LRRKR2/GBA).

Several lines of basic experimental data demonstrate convergent deleterious associations of *LRRK2* and *GBA* variations.^12,13^ These are consistent with limited clinical reports suggesting earlier age at onset of PD and increased penetrance in carriers of *LRRK2*/*GBA*.^14,15^ However, cross-sectional analyses of patients with established PD with both *LRRK2* and *GBA* variations suggest that carriers of *LRRK2*/*GBA* may instead have a less severe clinical course than those harboring *GBA* variations only,^15,16^ including less severe motor symptoms, and less dementia, and less severe nonmotor features in *LRRK2*/*GBA* PD compared with *GBA* PD.^15,16^ Because there are founder variations for *LRRK2* G2019S and *GBA* variations in individuals of Ashkenazi Jewish descent, the likelihood of identifying carriers of *LRRK2*/*GBA* is increased in this ethnic group. We expanded on these studies by evaluating longitudinal PD progression, with particular focus on continuous cognitive and motor scores in individuals with *LRRK2/GBA* PD compared with those with *LRRK2* PD, *GBA* PD, and idiopathic PD (ie, individuals without *GBA* or *LRRK2* variations), and assessing the *LRRK2* × *GBA* gene interaction on cognitive and motor decline. In addition, we extended the sample to include cohorts with individuals not of Ashkenazi Jewish descent as well.

## Methods

Ethical approval for the Mount Sinai Beth Israel (MSBI) Biomarker study was received from the Mount Sinai institutional review board, and all other sites and studies had local institutional review board approval. All participants provided written informed consent. This study is reported following the Strengthening the Reporting of Observational Studies in Epidemiology (STROBE) reporting guideline for cohort studies.

### Study Design

To maximize the number of dual *LRRK2* G2019S and *GBA* variant carriers with PD, we combined data from subgroups of prospective longitudinal cohorts of participants with or without Ashkenazi Jewish ancestry and included *LRRK2*/*GBA* PD and compared these with *LRRK2* PD, *GBA* PD, and idiopathic PD.

### Participants

Data from MSBI Biomarker study, National Institute of Neurological Disorders and Stroke (NINDS) Parkinson Disease Biomarker Program (PDBP),^17^ the Harvard Biomarkers Study (HBS),^18,19,20^ the Michael J. Fox Foundation (MJFF), *LRRK2* Ashkenazi Jewish Cohort Consortium (AJLCC, including Columbia, Tel Aviv Sourasky Medical Center, and MSBI),^21^ the SPOT study,^22^ and Parkinson Progression Marker Initiative (PPMI)^23^ were included. Study visits were performed between February 25, 2004, and December 31, 2019, depending on the study. The goal was to include the maximum number of individuals with *LRRK2*/*GBA* PD. Participants were screened for *LRRK2* and *GBA* variations and were included if they completed either a motor rating (Movement Disorder Society–sponsored revision of the Unified Parkinson Disease Rating Scale–Part III [MDS-UPDRS-III],^24^ or the original unrevised version [UPDRS-III]^25^) or a cognitive task (Montreal Cognitive Assessment [MoCA]^26^ or Mini-Mental State Examination [MMSE]^27^), with demographic information available. MSBI and AJLCC participants were genotyped for *LRRK2* G2019S and the 11 most common *GBA* variations among individuals of Ashkenazi Jewish descent (ie, N370S, 84GG, IVS2 + 1, V394L, D409G, L444P, A456P, RecNcil, R496H, E326K, and T369M). The PDBP and PPMI studies genotyped participants via NeuroX-derived genotyping or the Immunochip Array, including *GBA* T369M, E326K, N370S, F298L, C62Y, K13R, F255Y, E150K, E427K, D419N, or A309V, and the HBS study identified variations through targeted next-generation sequencing as previously described.^28,29^ PPMI genetic data were queried May 2020.

Our comparison groups focused on the subset of *LRRK2* PD previously described for progression (AJLCC, including follow-up at MSBI), and the subset of *GBA* PD not previously reported from the PDBP and MSBI studies (*GBA* PD and idiopathic PD). To prevent any double representation of participant enrollment, only unique data from a single cohort were included if a participant was enrolled in multiple cohorts. A total of 534 individuals with *LRRK2* or idiopathic PD from MSBI and the AJ LCC cohort were also included in prior description of longitudinal progression,^30^ and 3 participants with *LRRK2*/*GBA* PD in the PPMI study were previously reported.^16^ *GBA* variants were categorized^31^ into 4 variation severity categories: severe (including 84GG, L444P, L444R, R120W, RecNcil, V394L, and biallelic), mild (including N370S and R496H), variant (including E326K and T369M), and wild type.

### Clinical Measures

Criteria for PD and clinical, demographic, motor, and nonmotor data were collected per each study’s protocol, as previously described^17,18,21,22,23^ (Table 1). Longitudinal visits occurred a mean (SD) of 12 (3) months apart. Data were harmonized according to published conversions, with discordant scales converted to a single scale for analysis: the UPDRS-III^32^ subsection score was converted to the MDS-UPDRS III^24,25^ for the MSBI and AJLCC cohorts, and continuous MMSE^27^ scores were converted to MoCA^26^ scores for the HBS cohort as previously described.^33^

**Table 1.** Baseline Clinical and Demographic Features

Characteristic overall	No. (%) (N = 1193)				*P* value^a^
	*GBA* PD (n = 128)	*LRRK2* PD (n = 155)	*LRRK2/GBA* PD (n = 21)	Idiopathic PD (n = 889)	
Age, mean (SD) y	64.4 (9.9)	68.4 (9.2)	65.7 (9.0)	66.6 (10.0)	.006
Age at PD onset, y^b^	57.5 (10.2)	60.1 (9.6)	59.6 (9.9)	61.1 (10.0)	.003
Duration of PD, y	6.9 (5.9)	8.3 (6.5)	6.0 (6.7)	5.5 (4.9)	<.001
Sex					
Women	58 (45.3)	78 (50.3)	12 (57.1)	342 (38.5)	.015
Men	70 (54.7)	77 (49.7)	9 (42.9)	547 (61.5)	
Cohort					
MSBI (n = 307)	55 (43.0)	56 (36.1)	6 (28.6)	190 (20.9)	NA
HBS (n = 66)	26 (20.3)	0	0	40 (4.4)	NA
PDBP (n = 417)	27 (21.1)	0	1 (4.8)	389 (42.8)	NA
LCC (n = 394)	20 (15.6)	99 (63.9)	5 (23.8)	290 (31.9)	NA
PPMI (n = 7)	0	0	7 (33.3)	0	NA
SPOT (n = 2)	0	0	2 (9.5)	0	NA
Levodopa equivalent dose	692.7 (551.1)	747.4 (545.1)	283.3 (262.2)	580.7 (489.7)	<.001^f^
MDS-UPDRS III^c^	26.4 (13.6)	24.7 (14.2)	26.1 (9.1)	24.5 (13.5)	.680
MoCA^c^	24.8 (4.8)	25.4 (3.1)	25.7 (2.6)	25.5 (3.6)	.383

Abbreviations: HBS, Harvard Biomarkers Study; LCC, *LRRK2* Cohort Consortium; MDS-UPDRS III, Movement Disorder Society–sponsored revision of the Unified Parkinson Disease Rating Scale, part III; MoCA, Montreal Cognitive Assessment; MSBI, Mount Sinai Beth Israel; NA, not applicable; PD, Parkinson disease; PDBP, Parkinson Disease Biomarker Program; PPMI, Parkinson disease Progression Marker Initiative.

^a^ *P* values correspond to test for equality among all groups.

^b^ Age at PD determined using age at onset when available and age at diagnoses otherwise.

^c^ Participant data was selected for inclusion if there was a complete motor rating (MDS-UPDRS III) or a cognitive task (MoCA) at any visit during their participation and demographic information (sex, age, and age at PD onset) available.

### Statistical Analysis

The primary goals were the evaluation of the differences in the longitudinal rate of change in cognition (MoCA) and motor function (MDS-UPDRS III) among idiopathic PD, *LRRK2* PD, *GBA* PD (all variant types included in the main analysis), and *LRRK2*/*GBA* PD. Linear mixed effects models with robust variance estimates using PD duration (from age of onset) as the time scale were used to examine the associations of *LRRK2* and *GBA* genotypes on the rate of decline in MoCA and MDS-UPDRS-III scores. The primary models included *LRRK2* G2019S and *GBA* genotypes and their interaction term as the primary independent variables. Cognitive and motor progressions were modeled adjusting for sex, age at baseline, PD duration at baseline, and cohort as fixed effects. Models included a participant-specific random effect to account for the correlation in repeated measurements within the same participant. In addition, motor progression models adjusted for levodopa equivalent dose and for presence of deep brain stimulation implants at time of visit. All analyses were performed using Stata statistical software version 16 (StataCorp). Because there is heterogeneity among *GBA* variants,^31,34^ sensitivity analyses excluding severe and biallelic *GBA* variation carriers were also performed. Individual waves with missing data were excluded. All tests were 2-sided, and *P* < .05 was considered significant. Data were analyzed from May to July 2020.

## Results

### Demographic Characteristics and Overall Comparisons

Among 1193 participants with PD (mean [SD] age, 66.6 [9.9] years; 490 [41.2%] women) included, 128 (10.7%) had *GBA* PD, 155 (13.0%) had *LRRK2* PD, 21 (1.8%) had *LRRK2*/*GBA* PD, and 889 (74.5%) had idiopathic PD. The mean (SD) duration of study participation was 2.85 (1.43) years.

Site information and baseline clinical and genotype characteristics are presented in Table 1. Participants with *LRRK2* PD were older at baseline assessment (mean [SD] age, 68.4 [9.2] years) compared with individuals with idiopathic PD (mean [SD] age, 66.5 [10.0] years; *P* = .03) or *GBA* PD (mean [SD] age, 64.8 [9.7] years; *P* < .001) but not *LRRK2*/*GBA* PD (mean [SD] age, 65.7 [9.0] years; *P* = .18). Similarly, participants with *LRRK2* PD had longer mean (SD) duration of motor symptoms at baseline assessment (8.3 [6.5] years) compared with participants with idiopathic PD (5.5 [4.9] years; *P* < .001) or *GBA* PD (6.9 [5.9] years; *P* = .046) but not *LRRK2*/*GBA* PD (6.0 [6.7] years; *P* = .13). Participants with *GBA* PD were younger at motor symptoms onset (mean [SD] age, 57.5 [10.2] years) than those with *LRRK2* PD (mean [SD] age, 60.1 [9.6] years), *LRRK2*/*GBA *PD (mean [SD] age, 59.6 [9.9]), or idiopathic PD (mean [SD] age, 61.1 [10.0] years). Idiopathic PD was less common in women (342 women of 889 participants [38.5%]) compared with *GBA* PD (58 women of 128 participants [45.3%]), *LRRK2* PD (78 women of 155 participants [50.3%]), and *LRRK2*/*GBA* PD (78 women of 155 participants [57.1%]). There were no baseline differences in MDS-UPDRS III or MoCA scores among groups.

Participants with *GBA* PD were less likely to have mild variations and more likely to have high-risk variations than participants with *LRRK2*/*GBA* PD (biallelic: 8 participants [6.3%] vs 1 participant [4.8%]; severe: 11 participants [8.6%] vs 2 participants [9.5%]; mild: 49 participants [38.3%] vs 16 participants [76.2%]; variant: 60 participants [46.9%] vs 2 participants [9.5%]; overall *P* = .01) (Table 2); however, when mild or high-risk variant and severe or biallelic variations were combined, the distribution in *GBA* PD did not differ from *LRRK2/GBA* PD (109 participants [85.2%] vs 18 participants [85.7%]; *P* = .62). Information regarding deep brain stimulation was collected in all but 1 cohort, and included 6 participants with idiopathic PD (0.7%), 6 participants with *GBA* PD (4.7%), 5 participants with *LRRK2* PD (3.2%), and 2 participants with *LRRK2*/*GBA* PD (9.5%).

**Table 2.** *GBA* Gene Variation and Variant Frequencies

Variation^a^	No. (%)			*P* value^b^
	All (n = 149)	*GBA* only (n = 128)	*LRRK2* and *GBA* (n = 21)	
Severe and biallelic	22 (14.8)	19 (14.8)	3 (14.3)	>.99
Severe	13 (8.7)	11 (8.6)	2 (9.5)	.99
84GG	5 (38.5)	3 (27.3)	2 (100)	
L444P	3 (24.2)	3 (27.3)	0	
L444R	1 (6.7)	1 (9.1)	0	
R120W	1 (6.7)	1 (9.1)	0	
RecNcil	1 (6.7)	1 (9.1)	0	
V394L	2 (13.3)	2 (18.2)	0	
Biallelic	9 (6.4)	8 (6.3)	1 (4.8)	.97
N370S/84GG	1 (11.1)	1 (12.5)	0	
N370S/N370S	1 (11.1)	1 (12.5)	0	
N370S/R496H	2 (22.2)	2 (25.0)	0	
N370S/RecNcil	5 (55.6)	4 (50.0)	1 (100)	
Mild and Variant	127 (85.2)	109 (85.2)	18 (85.7)	>.99
Mild	65 (43.6)	49 (38.3)	16 (76.2)	.001
N370S	60 (92.3)	45 (91.8)	15 (93.75)	
R496H	5 (7.7)	4 (8.2)	1 (6.25)	
Variant	62 (41.6)	60 (46.9)	2 (9.5)	.001
E326K	46 (74.2)	44 (73.3)	2 (100)	
E326K/E326K	1 (1.6)	1 (1.7)	0	
T369M	15 (32.6)	15 (25.0)	0	

^a^ Mount Sinai Beth Israel and *LRRK2* Ashkenazi Jewish Cohort Consortium participants were genotyped for both *LRRK2* G2019S and the 11 most common *GBA* variations among individuals of Ashkenazi Jewish descent (ie, N370S, 84GG, IVS2 + 1, V394L, D409G, L444P, A456P, RecNcil, R496H, E326K, or T369M). Parkinson Disease Biomarker Program and Parkinson Progression Marker Initiative genotyped participants via NeuroX-derived genotyping or the Immunochip Array and included *GBA* variations T369M, E326K, N370S, F298L, C62Y, K13R, F255Y, E150K, E427K, D419N, and A309V. The Harvard Biomarker Study identified variations through targeted next-generation sequencing as previously described.^28,29^

^b^ *P* values correspond to test for equality among all groups.

### Rates of Cognitive Decline

**Figure 1.**
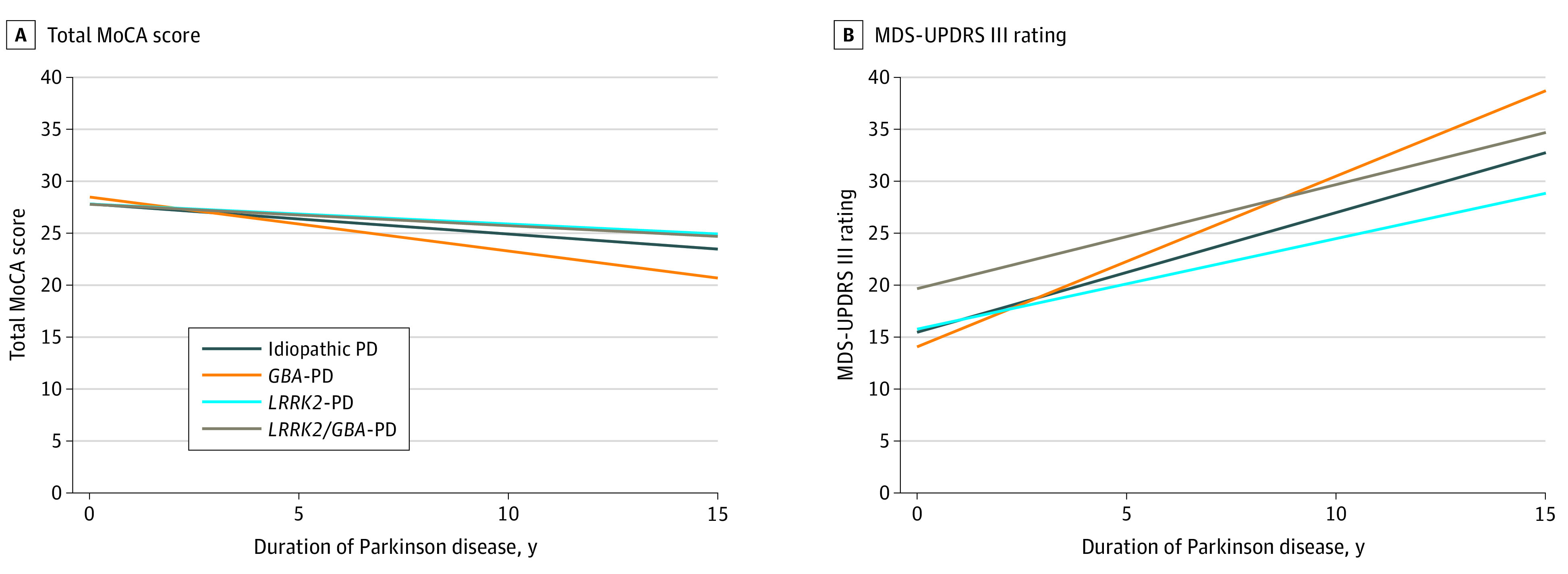
Longitudinal Trajectories of Mean Montreal Cognitive Assessment (MoCA) and Movement Disorders Society-Unified Parkinson Disease Rating Scale–Part III (MDS-UPDRS III) Rating Across Groups PD indicates Parkinson disease.

The estimated (SE) rates of decline in total MoCA score were −0.29 (0.05) points/y for idiopathic PD, −0.52 (0.09) points/y for *GBA* PD, −0.19 (0.06) points/y for *LRRK2* PD, and −0.21 (0.06) points/y for *LRRK2*/*GBA* PD (Figure 1). There was a slower, although not statistically significant, rate of cognitive decline for carriers with a *LRRK2* G2019S variation compared with individuals with idiopathic PD for *LRRK2* PD alone (B [SE], 0.10 [0.06] points/y; *P* = .08) and *LRRK2*/*GBA* PD (B [SE], 0.08 [0.05] points/y; *P* = .12). As anticipated, there was a worse rate of cognitive decline in participants with *GBA* PD compared with participants with idiopathic PD (B [SE], −0.23 [0.08] points/y; *P* = .005) and *LRRK2* PD (B [SE], −0.33 [0.09] points/y; *P* < .001), as well as those with *LRRK2*/*GBA* PD (B [SE], −0.31 [0.09] points/y; *P* < .001). However, there was no difference in rate of cognitive change between participants with *LRRK2* PD and those with *LRRK2*/*GBA* PD (B [SE], 0.01 [0.07] points/y; *P* = .85).

There was a significant *GBA* × *LRRK2* G2019S interaction in decline in cognitive scores (B [SE], 0.22 [0.11] points/y; *P* = .04). This interaction is described in detail in the eTable and eFigure in the Supplement. Specifically, among participants without the *LRRK2* G2019S variation, participants with *GBA* experienced faster decline compared with those without (ie, *GBA* PD vs idiopathic PD), but among participants with *LRRK2* G2019S, those with a *GBA* variation had no difference compared with participants without a *GBA* variation (ie, *LRRK2*/*GBA* PD vs *LRRK2* PD). Similarly, among participants without a *GBA* variation, carrying a *LRRK2* G2019S variation was associated with a slower cognitive decline (*LRRK2* PD vs idiopathic PD) as well as among individuals with *LRRK2/GBA* variations (*LRRK2*/*GBA* PD vs GBA PD). In sensitivity analysis excluding participants with severe *GBA* and dual variations the direction was the same, but the interaction was no longer significant (B [SE], 0.12 [0.11] points/y; *P* = .28).

### Rates of Motor Decline

The estimated (SE) rates of worsening in MDS-UPDRS III scores were 1.15 (0.16) points/y for idiopathic PD, 1.64 (0.26) points/y for *GBA* PD, 0.87 ± 0.20) points/y for *LRRK2* PD, and 1.00 (0.35) points/y for *LRRK2*/*GBA* PD. There was no significant difference in rate of motor change between participants with *LRRK2* PD and those with idiopathic PD or *LRRK2*/*GBA* PD, nor between participants with *LRRK2/GBA* PD and those with idiopathic PD. Participants with *GBA* PD had worse motor progression compared with those with idiopathic PD (estimate [SE], 0.49 [0.22] points/y; *P* = .03) and those with *LRRK2* PD (estimate [SE] 0.77 [0.26] points/y; *P* = .004), and a not statistically significant difference from those with *LRRK2*/*GBA* PD (estimate [SE], 0.64 [0.36] points/y; *P* = .07). There was no significant interaction association between *LRRK2* G2019S and *GBA* variations for motor progression in the main analysis (estimate [SE], −0.36 [0.40] points/y; *P* = .38) (Table 3).

**Table 3.** Models Comparing Rate of Change in MoCA Score and MDS-UPDRS III Among PD (Idiopathic PD, *GBA *PD, *LRRK2* PD, and *LRRK2*/*GBA* PD)

Characteristic	MoCA		MDS-UPDRS III^a^	
	B (SE)	*P* value	B (SE)	*P* value
Age at baseline, per y	−0.13 (0.01)	<.001	0.36 (0.04)	<.001
Men (vs women)	−0.66 (0.19)	<.001	3.95 (0.78)	<.001
Site (vs PDBP)				
HBS	0.46 (0.32)	.15	379 (1.46)	.009
MSBI	0.01 (0.26)	.98	−3.54 (1.08)	.001
CUIMC LCC	0.60 (0.32)	.06	−0.08 (1.44)	.95
Tel Aviv LCC	−1.11 (0.28)	<.001	4.78 (1.14)	<.001
PPMI	−0.90 (0.95)	.35	−0.54 (3.90)	.89
CUIMC SPOT	0.66 (2.05)	.75	5.48 (9.09)	.55
Baseline duration, per y	0.14 (0.05)	.005	−0.37 (0.17)	.03
Baseline PD (vs idiopathic PD)				
* GBA*	0.66 (0.55)	.23	−1.40 (1.92)	.47
* LRRK2*	−0.01 (0.51)	.99	0.03 (1.90)	.99
* LRRK2/GBA*	−0.03 (0.67)	.96	4.19 (3.78)	.27
PD slope, points/y				
Idiopathic	−0.29 (0.05)	<.001	1.15 (0.16)	<.001
* GBA*	−0.52 (0.09)	<.001	1.64 (0.26)	<.001
* LRRK2*	−0.19 (0.06)	.001	0.87 (0.20)	<.001
*LRRK2*/*GBA*	−0.20 (0.06)	.001	1.0 (0.35)	.004
PD slope differences between groups, point/y				
*GBA* vs idiopathic	−0.23 (0.08)	.005	0.49 (0.22)	.03
*LRRK2* vs idiopathic	0.10 (0.06)	.08^b^	−0.28 (0.19)	.14
*LRRK2*/*GBA* vs idiopathic	0.08 (0.05)	.12	−0.15 (0.32)	.64
*GBA* vs *LRRK2*	−0.33 (0.09)	<.001	0.77 (0.26)	.004
*GBA* vs *LRRK2/GBA*	−0.31 (0.09)	<.001	0.64 (0.36)	.07
*LRRK2* vs *LRRK2/GBA*	0.01 (0.07)	.85	−0.13 (0.35)	.71
Interaction between *GBA* and *LRRK2* variation	0.22 (0.11)	.04	−0.36 (0.40)	.38

Abbreviations: CUIMC, Columbia University Irving Medical Center; HBS, Harvard Biomarkers Study; LCC, *LRRK2* Cohort Consortium; MDS-UPDRS-III, Movement Disorder Society sponsored revision of the Unified Parkinson Disease Rating Scale, part III; MoCA, Montreal Cognitive Assessment; MSBI, Mount Sinai Beth Israel; PD, Parkinson disease; PDBP, Parkinson Disease Biomarker Program.

^a^ For the MDS-UPDRS-III only model, in addition to the covariates listed, levodopa equivalent dose (per 100 mg) and deep brain stimulation therapy received at the time of the visit as fixed-effects covariates were also included. The association of levodopa equivalent dose with progression was estimated in the model as B (SE), −0.13 (0.07) points/y (*P* = .07) while DBS was estimated as B (SE), −7.75 (3.87) points/y (*P* = .045).

^b^ Tests for the presence of an interaction of variation type (*LRRK2* variations and *GBA* variations) in the rate of cognitive progression (MoCA) and motor progression (MDS-UPDRS III) were performed based on slope estimates from the main models. In secondary models limited to sites of enrollment including participants of Ashkenazi Jewish descent, B (SE) was 0.19 (0.15) points/y (*P* = .20). In models adjusting for education B (SE) was 0.19 (0.11) points/y (*P* = .08). In models excluding participants with severe *GBA* and biallelic variations B (SE) was 0.12 (0.11) points/y (*P* = .28). In models limited to mild *GBA* variations, B (SE) was 0.07 (0.16) points/y (*P* = .64). In these models, the *LRRK2* × *GBA* interactions for decline in cognitive scores were no longer significant. Furthermore, in a final secondary model also adjusting for level of education, the interaction was no longer significant (B [SE], 0.19 [0.11] points/y; *P* = .08). Of note, while the difference between *LRRK2* PD and idiopathic PD did not reach statistical significance here (difference estimate [SE], −0.281 [0.19] points/y; *P* = .14), the magnitude and direction of the difference was similar to our prior work.^30^

## Discussion

In this cohort study systematically evaluating longitudinal cognitive scores, *LRRK2* G2019S and *GBA* variations did not have a combined deleterious association with disease progression in participants with both mutations. Cognitive decline in participants with *LRRK2*/*GBA* PD was less severe than in those with *GBA* PD and more closely resembled that observed in participants with *LRRK2* PD. Our findings are overall consistent with those of 2 major studies evaluating *LRRK2*/*GBA*^15,16^ and are in alignment by finding that nonmotor anomalies were less prominent in individuals with *LRRK2*/*GBA* variations than in those with *GBA* variations. In a 2019 study by Yahalom et al in an Ashkenazi Jewish cohort,^15^ individuals with *LRRK2*/*GBA* PD had significantly less cognitive impairment, more rapid eye movement sleep behavior disorder, and less dementia and psychosis than those with *GBA* PD. In a larger and separate Israeli study evaluating individuals with *LRRK2*/*GBA* PD,^16^ there were no cross-sectional differences in MoCA performance, but there was less depression in participants with *LRRK2*/*GBA* PD than among those with severe variation *GBA* PD and more preserved olfaction in participants with *LRRK2*/*GBA* PD, as well as greater motor function than in those with *GBA* PD. Omer et al^16^ suggested a dominant association of the *LRRK2* variation in participants with variations in *LRRK2* and *GBA*. Our work extends these prior studies^15,16^ through longitudinal evaluation of continuous measures of cognition and motor function and with inclusion of participants with and without Ashkenazi ancestry. We specifically demonstrated that the longitudinal cognitive decline in individuals with *LRRK2*/*GBA* PD, as measured by MoCA, was more similar to that observed in individuals with *LRRK2* PD, consistent with the cross-sectional data from the study by Yahalom et al.^15^

Our study also expands prior work by identifying a novel statistical interaction between *LRRK2* G2019S variations and *GBA* variations in cognitive decline. However, we cannot be certain that the statistical interaction translates to a biological one. Genetic heterogeneity, including *GBA* allelic differences, as well as differences in *GBA* and *LRRK2* penetrance, clinical expression, and distribution of types of variations in the *GBA* and *LRRK2/GBA* groups may all play roles in explaining the statistical interaction. Sensitivity analyses excluding individuals with severe *GBA* variations no longer demonstrated significance in the interaction association for cognitive decline. However, it should be noted that the power to detect an interaction was subsequently reduced in this restricted sensitivity sample. Furthermore, although the magnitude of the association was reduced, the direction of the association was maintained, suggesting that the overall association of *GBA* is still diminished in those with the *LRRK2*/*GBA* variation. In a 2020 cross-sectional evaluation of the largest sample of individuals with *LRRK2*/*GBA* dual variation,^16^ the dominant association of *LRRK2 *G2019S persisted with analysis restricted to individuals with mild *GBA* variations.

As anticipated from prior studies of single variation data,^35,36^ we found that harboring a *GBA* variation was associated with faster motor deterioration. While the slope of motor decline was worse in participants with *GBA* PD than in those with *LRRK2*/*GBA*PD, the difference was not statistically significant. The difference between participants with *LRRK2* PD and those with idiopathic PD also did not reach statistical significance, but the magnitude and direction of the difference was similar to our prior work.^30^

Our overall finding, that cognition was not worse in participants with *LRRK2*/*GBA* PD than in those with only the *LRRK2* variation, challenges cellular and clinical data suggesting convergent deleterious associations of dual *LRRK2* and *GBA* variations. Studies in *GBA1* variant mouse astrocytes^13^ and in human *LRRK2* variation–induced pluripotent stem cell^12^ derived dopamine neurons support a convergent deleterious association of *GBA* and *LRRK2* variations on some phenotypes; that is, inhibiting the overactivity of LRRK2 kinase improved function in the lysosomal glucocerebrosidase *GBA* pathway and ameliorated *GBA* variation–mediated cell loss. However, these findings are in contrast to the elevated peripheral glucocerebrosidase activity observed from dried blood spots from individuals with *LRRK2* G2019S PD.^22^

**Figure 2.**
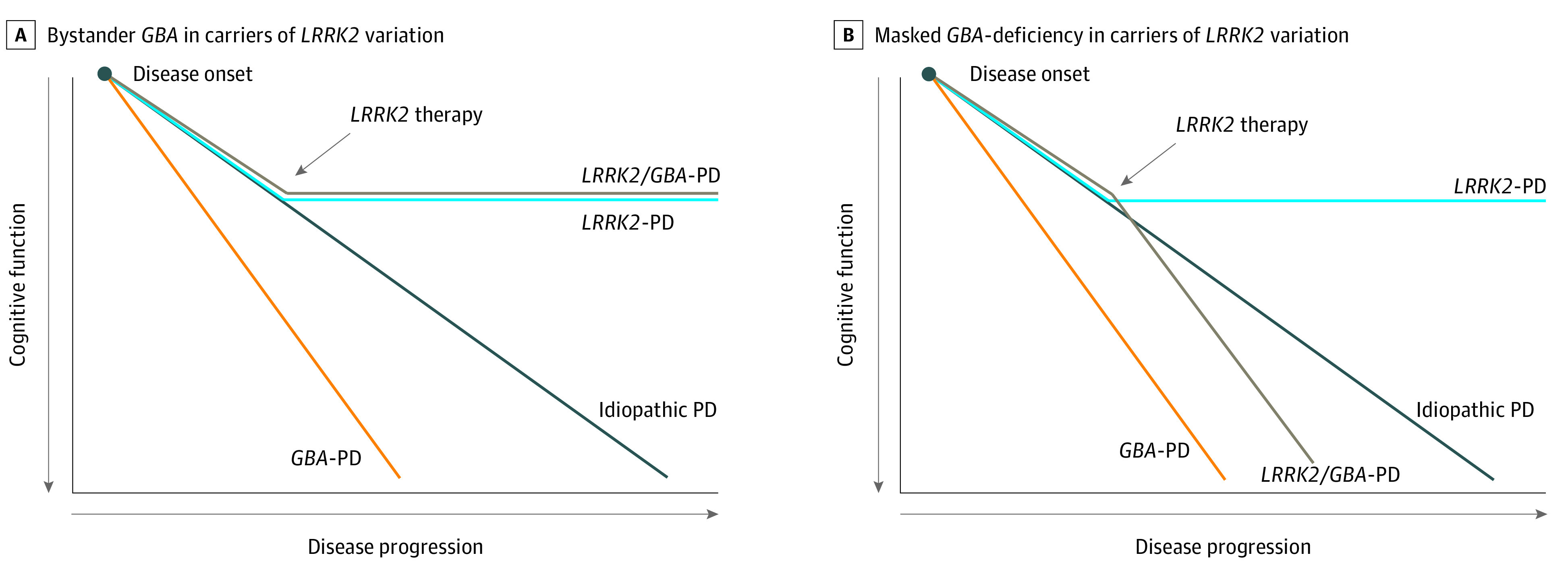
Hypothetical Outcomes in *LRRK2*-Targeted Therapies in Different Genetic Groups After disease onset, in the case that *GBA* mutations are benign in *LRRK2* Parkinson disease (PD), according to an inert hypothesis for *LRRK2*/*GBA* carriers, treatment responses for effective *LRRK2*-targeting therapies would be identical for carriers of *LRRK2* and *LRRK2*/*GBA* carriers. Alternatively, in the masked hypothesis for carriers of *LRRK2*/*GBA*, *LRRK2* variations are biologically masking the effects of *GBA* variations in disease progression, and *LRRK2*-targeting therapy would unmask the effects of GBA variations to match disease progression and cognitive decline associated with *GBA* variations.

Furthermore, not all studies that evaluated PD penetrance and age at onset among individuals of Ashkenazi Jewish descent with PD suggested a combined deleterious association in carriers of both *LRRK2* G2019S and *GBA* variations or risk variants. Some have demonstrated a higher odds ratio of developing PD, as well as an earlier age of PD onset, than in dual carriers vs those of only a *LRRK2 *G2019S or *GBA* variant.^14,15^ If these penetrance and age at onset findings are replicated, one explanation that may reconcile these with the finding of less severe nonmotor progression in carriers of dual *LRRK2*/*GBA* variations is that *LRRK2* and *GBA* may differentially be associated with the development of PD (penetrance and onset) and the PD disease course (clinical course). We speculate that once PD is established, the joint association might occur in 1 of 2 ways. On the one hand, individuals with dual *LRRK2*/*GBA* variations might be more likely to express *LRRK2-*associated pathologic and clinical expression when *LRRK2* G2019S variations and associated gain-of-function renders otherwise deleterious *GBA* variations inert with respect to *GBA* progression phenotypes (Figure 2). Alternatively, because most *GBA* variations have significantly lower penetrance than *LRRK2* G2019S, *GBA* variations in these individuals with *LRRK2*/*GBA* variations may be nonpenetrant carriers, and the clinical course may be driven by the *LRRK2* variation, with the *GBA* variation not demonstrating great association, particularly for mild or high-risk variations. However, the biological underpinnings of these possibilities are not clear, and further interrogation of biological mechanisms, including biomarker analysis in a larger sample of individuals with PD harboring dual *LRRK2* and severe *GBA* variations, and an analysis of the consequence of *LRRK2* and/or *GBA* variations on such a biomarker is needed.

A strength of our study is that we have pooled and analyzed standardized, prospective data with up to 7 years of follow-up. Unlike previous studies that have published data on individuals with *LRRK2/GBA*, our analysis allowed longitudinal assessment to estimate the rate of motor and cognitive progression in *LRRK2/GBA* PD. Furthermore, to increase the sample size and enrich the genetic cohorts, our study included data collected from multiple cohorts and increased the proportion and variation of *GBA* variation types represented. However, because Ashkenazi Jewish ethnicity was not queried in 2 sites, we could not stratify the entire study. In sensitivity analysis limited to individuals with known Ashkenazi Jewish ethnicities, the slope of cognitive decline in *LRRK2/GBA* PD remained less than *GBA PD*.

### Limitations

Our study has some weaknesses, such as the noted genetic heterogeneity across sites and variation groups, including the few individuals with dual *LRRK2/GBA* variation and severe *GBA* variations, and the small sample size of individuals with *LRRK2/GBA* PD, which limits generalizability. As we did not have equivalent *GBA* sequencing data for all sites, some individuals with *GBA* variations were likely miscoded as having idiopathic PD. However, this would bias toward the null rather than toward our finding in cognition. Not all data were available or used from all cohorts. For example, HBS data were limited to those associated with the MSBI study, and to avoid any potential overlap, we limited PPMI data to *LRRK2*/*GBA* PD and did not include the entire PPMI cohort data. In sensitivity analyses excluding the 7 individuals with dual *LRRK2/GBA* variation from PPMI, all findings, including group differences in the rate of change in MoCA score and MDS-UPDRS III rating, and an interaction association in MoCA were maintained. Additionally, there was a need to harmonize measures across sites, and furthermore, MoCA scores may be lower in non-English versions.^37^

## Conclusions

The findings of this cohort study support prior, primarily cross-sectional data, suggesting that individuals with dual *LRRK2*/*GBA* variations do not have a worse PD clinical course than individuals with *GBA* PD or *LRRK2* PD alone. We also found a statistical interaction between *LRRK2* G2019S and *GBA* that may point to a possible biological association of *LRRK2* G2019S canceling the deleterious associations of *GBA*. However, because the association was no longer significant in sensitivity analyses excluding individuals with severe or dual variations, we cannot exclude that the interaction may be a statistical reflection of genetic heterogeneity in this group, and that the differential associations of *GBA* variations and the interaction’s biological relevance are not certain. Because it was challenging to identify individuals that harbor both *GBA* and *LRRK2* G2019S variations and we combined diverse groups, further investigation with systematic larger groups, especially including individuals with severe *GBA* variations, is warranted. This will facilitate better understanding of putative interaction mechanisms and potential therapeutic implications for cognitive progression.

## Notes

Supplement. **eTable.** Interaction Between *LRRK2* and *GBA* for Rate of MoCA Decline **eFigure.** Interaction Between *LRRK2* and *GBA* for Rate of MoCA Decline
