# Supplementary material for: Association of Dual LRRK2 G2019S and GBA Variations With Parkinson Disease Progression
Source: JAMA Netw Open. 2021 Apr 21;4(4):e215845. doi: 10.1001/jamanetworkopen.2021.5845 (PMC8060834; doi:10.1001/jamanetworkopen.2021.5845)
Supplement: Supplement. — eTable. Interaction Between LRRK2 and GBA for Rate of MoCA Decline eFigure. Interaction Between LRRK2 and GBA for Rate of MoCA Decline [file jamanetwopen-e215845-s001.pdf]

Supplemental Online Content

Ortega RA, Wang C, Raymond D, et al. Association of dual *LRRK2* G2019S and *GBA* variation with Parkinson disease progression. *JAMA Netw Open*. 2021;4(4):e215845. doi:10.1001/jamanetworkopen.2021.5845

eTable. Interaction Between *LRRK2* and *GBA* for Rate of MoCA Decline
eFigure. Interaction Between *LRRK2* and *GBA* for Rate of MoCA Decline

This supplemental material has been provided by the authors to give readers additional information about their work.

© 2021 Ortega RA et al. *JAMA Network Open.*

eTable. Interaction Between *LRRK2* and *GBA* for Rate of MoCA Decline

	Estimate: B ± SE[1]
Interaction effect between *LRRK2* and *GBA* mutations on the rate of cognitive progression*:<br><br>B minus A**:<br>Difference in presence of *LRRK2* - difference in absence of *LRRK2*<br>** As well as D minus C:<br>Difference in presence of *GBA* – difference in absence of *GBA*	 <br><br>0.22 ± 0.11 points/year<br>p=0.044
Effect of *GBA* mutations on non-*LRRK2*-G2019S carriers:<br>Decline among *GBA*-PD<br>Decline among idiopathic PD<br><br>A: Difference in absence of *LRRK2*-G2019S:<br>*GBA*-PD minus idiopathic PD	 -0.52 ± 0.09 points/year<br>-0.29 ± 0.05 points/year<br><br>-0.23 ± 0.08 points/year<br>p=0.005
Effect of *GBA* mutations on *LRRK2*-G2019S carriers:<br>Decline among *LRRK2/GBA*-PD<br>Decline among *LRRK2*-PD<br><br>B: Difference in presence of *LRRK2*-G2019S:<br>*LRRK2/GBA*-PD minus *LRRK2*-PD	 -0.21 ± 0.06 points/year<br>-0.19 ± 0.06 points/year<br><br>0.01 ± 0.07 points/year<br>p=0.845
Effect of *LRRK2* mutations on non-*GBA* carriers<br>Decline among *LRRK2*-PD<br>Decline among idiopathic PD<br><br>C: Difference in absence of *GBA*:	 -0.21 ± 0.06 points/year<br>-0.29 ± 0.05 points/year<br><br>0.10 ± 0.06 points/year

© 2021 Ortega RA et al. *JAMA Network Open.*

LRRK2-PD minus idiopathic PD	p=0.080
Effect of *LRRK2* mutations on *GBA* carriers:<br>Decline among *LRRK2*/*GBA*-PD<br>Decline among *GBA*-PD<br><br>D: Difference in presence of *GBA-PD*:<br>*LRRK2*/*GBA*-PD minus *GBA*-PD	 -0.21 ± 0.06 points/year<br>-0.52 ± 0.09 points/year<br><br>0.31 ± 0.092 points/year<br>p<0.001

[1] This table demonstrates the requisite estimates involved in deriving the interaction effect between *LRRK2* and *GBA* mutations on the rate of cognitive progression. The interaction effect can be estimated by subtracting the effect of harboring a *GBA* mutation on rate of cognitive decline among non-*LRRK2*-G2019S mutation carriers (e.g. slopes in *GBA*-PD minus idiopathic PD) from the effect of harboring a *GBA* mutation on the rate of cognitive decline among *LRRK2*-G2019S mutation carriers (e.g. slopes in *LRRK2*/*GBA*-PD minus *LRRK2*-PD). Similarly, it could be estimated by subtracting the effect of harboring a *LRRK2* mutation on rate of cognitive decline among non-*GBA* mutation carriers (e.g. slopes in *LRRK2*-PD minus idiopathic PD) from the effect of harboring a *LRRK2* mutation on the rate of cognitive decline among *GBA* mutation carriers (e.g. slopes in *LRRK2*/*GBA*-PD minus *GBA*-PD).

eFigure. Interaction Between *LRRK2* and *GBA* for Rate of MoCA Decline

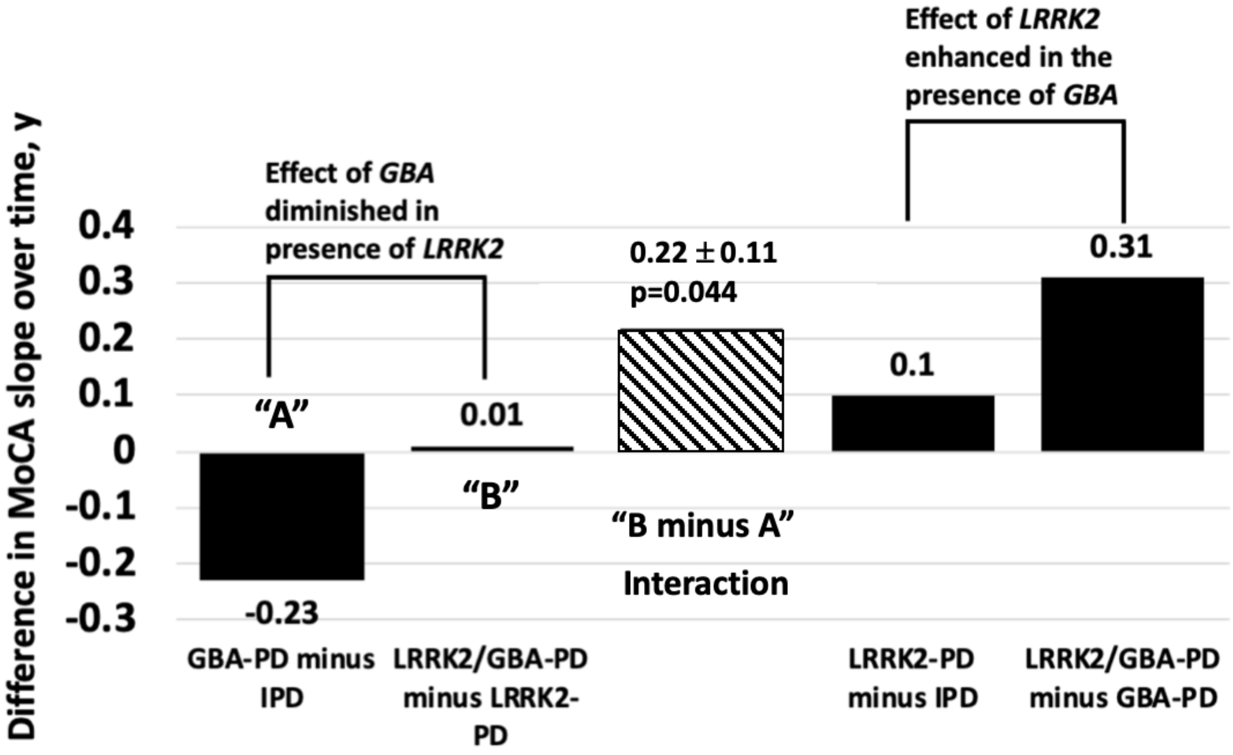

eFigure 1. The interaction effect can be estimated by subtracting the effect of harboring a *GBA* mutation on rate of cognitive decline among non-*LRRK2* mutation carriers (e.g. slopes in *GBA*-PD minus idiopathic PD) from the effect of harboring a *GBA* mutation on the rate of cognitive decline among *LRRK2* mutation carriers (e.g. slopes in *LRRK2*/*GBA*-PD minus *LRRK2*-PD). Similarly, it could be estimated by subtracting the effect of harboring a *LRRK2* mutation on rate of cognitive decline among non-*GBA* mutation carriers (e.g. slopes in *LRRK2*-PD minus idiopathic PD) from the effect of harboring a *LRRK2* mutation on the rate of cognitive decline among *GBA* mutation carriers (e.g. slopes in *LRRK2*/*GBA*-PD minus *GBA*-PD). See also Supplemental table 1.

© 2021 Ortega RA et al. *JAMA Network Open.*

© 2021 Ortega RA et al. *JAMA Network Open.*
